# Fitbit Use and Activity Levels From Intervention to 2 Years After: Secondary Analysis of a Randomized Controlled Trial

**DOI:** 10.2196/37086

**Published:** 2022-06-30

**Authors:** Sheri J Hartman, Ruohui Chen, Rowena M Tam, Hari K Narayan, Loki Natarajan, Lin Liu

**Affiliations:** 1 Herbert Wertheim School of Public Health and Human Longevity Science University of California, San Diego La Jolla, CA United States; 2 Moores Cancer Center University of California, San Diego San Diego, CA United States; 3 Department of Pediatrics University of California, San Diego San Diego, CA United States

**Keywords:** physical activity, fitness, exercise, Fitbit, wearable, health technology, mHealth, digital health, activity tracker, maintenance, adherence, tracker, use pattern, activity level, behavior change, cancer, breast, survivor, long-term use, sustained use

## Abstract

**Background:**

There has been a rapid increase in the use of commercially available activity trackers, such as Fitbit, in physical activity intervention research. However, little is known about the long-term sustained use of trackers and behavior change after short-term interventions.

**Objective:**

This study aims to use minute-level data collected from a Fitbit tracker for up to 2 years after the end of a randomized controlled trial to examine patterns of Fitbit use and activity over time.

**Methods:**

Participants in this secondary data analysis were 75 female breast cancer survivors who had been enrolled in a 12-week physical activity randomized controlled trial. Participants randomized to the exercise intervention (full intervention arm) received a Fitbit One, which was worn daily throughout the 12-week intervention, and then were followed for 2 years after the intervention. Participants randomized to the waitlist arm, after completing the randomized controlled trial, received a Fitbit One and a minimal version of the exercise intervention (light intervention arm), and then were followed for 2 years after the intervention. Average and daily adherence and MVPA were compared between the 2 groups in the interventional and postinterventional periods using both linear and generalized additive mixed effects models.

**Results:**

Adherence to wearing the Fitbit during the 12-week intervention period was significantly higher in the full intervention arm than in the light intervention arm (85% vs 60%; *P*<.001). Average adherence was significantly lower for both study arms during the follow-up period than in the intervention period; however, there were statistically different patterns of adherence during the follow-up period, with the light intervention arm having steeper declines than the full intervention arm over time (*P*<.001). Similar to the adherence results, mean minutes of Fitbit-measured MVPA was higher for the full intervention arm than for the light intervention arm during the 12-week intervention period (mean MVPA 27.89 minutes/day, SD 16.38 minutes/day vs 18.35 minutes/day, SD 12.64 minutes/day; *P*<.001). During the follow-up period, average MVPA was significantly lower than the 12-week intervention period for both the full intervention arm (21.74 minutes/day, SD 24.65 minutes/day; *P*=.002) and the light intervention arm (15.03 minutes/day, SD 13.27 minutes/day; *P*=.004). Although the mean MVPA in each arm was similar across the follow-up period (*P*=.33), the pattern of daily MVPA was significantly different between the 2 groups (*P*<.001).

**Conclusions:**

While adherence to wearing activity trackers and maintaining physical activities declined after completion of a 12-week exercise intervention, a more active interventional strategy resulted in greater wear time and activity levels during the intervention and more stable patterns of adherence and activity in the long term. An improved understanding of long-term maintenance patterns may inform improved exercise interventions that result in sustained increases in physical activity.

**Trial Registration:**

ClinicalTrials.gov NCT02332876; https://clinicaltrials.gov/ct2/show/NCT02332876

## Introduction

There are currently 3.9 million breast cancer survivors in the United States; most of whom do not engage in sufficient physical activity to meet current recommendations [1]. Greater physical activity in breast cancer survivors is associated with better quality of life, lower risk of all-cause and breast cancer–specific mortality, and lower risk of recurrent breast cancer [2-6], but 34% of cancer survivors report no physical activity in their leisure time [7,8]. An abundance of evidence demonstrates that interventions to increase physical activity in breast cancer survivors can be effective in the short term [4,5]. However, there are few studies examining maintenance of longer-term physical activity behavior beyond the intervention period [9] and those that do suggest that physical activity declines over time [10-12]. An improved understanding of maintenance behaviors is needed to optimize interventions to sustain increases in physical activity over the long term. Wearable trackers, such as Fitbit, capture physical activity behaviors and provide self-monitoring feedback, thereby offering both greater insight into maintenance behaviors and a potential method to facilitate sustained improvements in long-term maintenance.

Self-monitoring is one of the key skills to promote behavior changes [13], and may have a role in promoting sustained increases in physical activity in breast cancer survivors. The behavior change techniques framework proposed by Michie and colleagues [13,14] suggests that self-monitoring is the skill most strongly associated with intervention success when combined with at least one other self-regulatory technique from Control Theory (eg, receiving feedback on performance and reviewing progress toward goals) [15,16]. According to Control Theory, feedback loops provide awareness of discrepancies between performance and goals that can encourage behavior change [15]. Wearable trackers facilitate self-monitoring and feedback loops by passively collecting and providing information and feedback on progress toward individual goals.

Initial studies on Fitbit adoption have demonstrated that they are effective in increasing physical activity levels when coupled with other interventions [17-21], but the novelty of wearing the tracker wears off over time [22]. Additionally, prior studies have either been short term or had continued contacts with the participants in their maintenance phase [22-24]. Studies that have only utilized Fitbit as a means of behavior change show no significant changes in physical activity [25-27]. This decline in novelty, short interventional period, and variable additional support throughout the intervention may negatively affect use of the wearable technology when external accountability from the research study is removed [28-31].

This analysis explored adherence to wearing the Fitbit and physical activity 2 years after the end of a 3-month randomized controlled trial comparing a physical activity intervention (full intervention arm) with a waitlist control that received a “light” intervention (light intervention arm) after completing the 12-week assessments [24]. The aims of this study were to (1) examine patterns of adherence to wearing the Fitbit between the full intervention arm and the light intervention arm during their respective 3-month intervention periods and up to 2 years’ follow-up; (2) examine patterns of Fitbit-measured physical activity between the full intervention arm and the light intervention arm during their respective 3-month intervention periods and up to 2 years’ follow-up.

## Methods

### Participants and Design

Participants in this secondary data analysis were originally randomized to a 12-week physical activity intervention group or a waitlist control group. After completing final measures for the randomized trial at week 12, participants were invited to enroll in a maintenance study where their Fitbit data would be collected for the next 2 years and they would complete online questionnaires every 6 months over the next 2 years (4 times total). Participants were asked to provide written informed consent for participating in the maintenance study. Data from the original randomized trial and the 2-year follow-up were collected from February 2015 to July 2018. The intervention trial was registered with Clinicaltrials.gov (NCT02332876).

Eligible participants were female breast cancer survivors, aged 21-85 years, who were diagnosed less than 5 years prior to study enrollment, had completed chemotherapy or radiation treatment, were sedentary (defined as self-reporting <60 minutes of moderate-to-vigorous physical activity [MVPA] in 10-minute bouts per week), and had access to the internet and a Fitbit-compatible computer, tablet, or phone. Exclusion criteria included any medical condition that could make it potentially unsafe to be in an unsupervised physical activity intervention (determined by the Physical Activity Readiness Questionnaire [32]), other primary or recurrent invasive cancer within the last 10 years, and inability to commit to a 12-week intervention. All participants who returned for the 12-week assessment were eligible to enroll in the maintenance study.

A detailed description of the original trial’s protocol was previously published [33]. Briefly, potential participants were telephone screened, with interested and eligible women scheduled for an in-person visit to provide signed informed consent and complete baseline measures. Participants returned about 1 week later for their second visit where they were randomly assigned to 1 of 2 groups, an exercise intervention or waitlist control, in a 1:1 ratio. After randomization, participants in both groups reviewed the expectations and requirements of their group assignment with study staff.

### Physical Activity Intervention (Full Intervention Arm)

Participants randomized to the full intervention arm had a 30- to 45-minute in-person meeting where they went on a 10-minute walk at moderate intensity and set personalized physical activity goals with a researcher trained in motivational interviewing aimed at gradually working up to 150 minutes/week of MVPA. Participants were given a Fitbit One (Fitbit, Inc./Google) to self-monitor their physical activity, set up the Fitbit with their coach, taught how to use it, and taken on a 10-minute walk. Participants were also informed that their health coach would be reviewing their Fitbit activity data weekly and that they would receive feedback on the Fitbit data during the scheduled phone calls and between calls as needed. Participants received 2 scheduled phone calls (2- and 6-week time points) and emails every 3 days throughout the 12-week intervention. The intervention was delivered by a clinical psychologist with extensive training and experience in promoting behavior change (SJH) and by a staff member who was trained by SJH. For further details on the intervention, see Hartman et al [33]. No additional intervention content and support were received during the 2-year follow-up period.

### Waitlist Plus Light Physical Activity Intervention (Light Intervention Arm)

After completion of measures at the final visit, participants in the light intervention arm were provided with a “light” version of the exercise intervention. In a 15-20-minute in-person meeting, participants in the light intervention arm worked with a measurement research assistant to set personalized physical activity goals. The research assistant had received training on goal setting from SJH, with a brief introduction to using motivational interviewing. Participants received the Fitbit One with instructions on how to use it to support self-monitoring. Different from the full intervention arm, participants did not set up the Fitbit with their health coach, they were not told that their health coach could see their data nor that they would receive any feedback on their Fitbit data. Participants were also not taken on the 10-minute walk to demonstrate moderate intensity. Participants were offered the same 2 phone calls (2 and 6 weeks later), but these calls were framed as optional. Participants received the same automated emails every 3 days for the next 12 weeks that the full intervention arm received.

### Two-Year Maintenance Study Assessments

At the completion of their respective intervention, participants in both arms were asked to sync and charge their Fitbit at least once per week. When participants had not synced their Fitbit for 2 weeks, study staff would contact them to ask them to sync and provide any tech support if there were challenges syncing. Participants also received 4 online questionnaires to complete every 6 months across the 2-year follow-up.

### Measures

The Fitbit One, a commercially available accelerometer-based activity tracker, was used to examine patterns of physical activity throughout the 12-week intervention. Fitbit uses a proprietary algorithm to classify each minute as being in sedentary, light, moderate, or vigorous activity, and provides metabolic equivalents (METs) for each minute. Data were wirelessly uploaded to the user’s Fitbit account online and then downloaded by the research team through a database called Fitabase (Small Steps Lab), which allows for collecting data at the minute level. Daily adherence to wearing the Fitbit tracker was defined as wearing the tracker for over 10 hours in a day or logging at least some activity (over 1 minute of MVPA). This definition for a valid Fitbit wear day was used because participants were not instructed to wear the Fitbit all day; rather they were instructed to use the Fitbit to track activity. Thus, wearing the tracker specifically to log MVPA was deemed to be valid wear based on these instructions. Fitbit wear time was determined by processing of minute-level Fitbit data using the R function *accel.weartime* within the “accelerometry” package [34]. Nonwear was classified using both steps and METs. Consistent with standard protocols for ActiGraph accelerometry wear time [35], greater than 90 consecutive minutes of 0 steps/METs with 2-minute tolerance (ie, for 2 minutes with nonzero counts during nonwear intervals) was deemed nonwear.

Both groups wore the ActiGraph for 7 days prior to receiving the Fitbit and starting the full or light intervention. The ActiGraph GT3X+, a well-validated research-grade accelerometer [36], provided frequency, duration, and intensity of physical activity. Using standard guidelines, sufficient ActiGraph wear time was classified as over 10 hours of wear a day for at least 5 days or over 50 hours across 4 days and screened for in the ActiLife software using guidelines outlined by Choi et al [35]. All complete and valid data were processed in the ActiLife software using the low-frequency extension and aggregated to 60-second epochs so that published physical activity cut points could be applied [37]. MVPA was defined as 1952 or more counts per minute (3.00-7.00 METs). The full intervention arm wore the Fitbit and ActiGraph concurrently for 7 days to assess validity of Fitbit-measured MVPA. Fitbit-measured MVPA was highly correlated with ActiGraph-measured MVPA collected on overlapping days (r=0.81: ActiGraph MVPA/day mean 29.9 minutes, SD 25.90 minutes; Fitbit MVPA/day mean 25.8 minutes, SD 28.76 minutes), as we have previously reported [24].

On the questionnaire administered at 6, 12, 18, and 24 months, participants were asked if they were still wearing their Fitbit. If they reported they were not wearing it, they were asked the reason they stopped wearing the Fitbit.

### Statistical Analysis

Participants who did not consent for 2-year maintenance study were excluded from the analysis. Group differences in baseline characteristics between those who consented to the 2-year study and those who did not were assessed using 2-sample independent *t* test (unpaired) and chi-square test. Baseline characteristics were summarized between the full intervention arm and the light intervention arm.

### Adherence to Wearing the Fitbit and Daily MVPA During the 12-Week Intervention Period and 2-Year Follow-Up

The mean weekly rolling average adherence to wearing the Fitbit and mean MVPA were calculated by averaging the outcomes over the first 12-week period and over the 2-year follow-up period separately for each individual. Descriptive statistics and boxplots were used to summarize the adherence to wearing the Fitbit and MVPA at 12-week and 2-year follow-up as well as the change from 12 weeks to 2 years.

### Comparison of Adherence to Wearing the Fitbit and MVPA Between the Full Intervention Arm and the Light Intervention Arm

For comparing mean outcomes and mean change in outcomes between the 2 intervention groups, we used the following linear mixed effects model:

E(Y) = *β*_0_ + *β*_1_ × Arm + *β*_2_ × Period + *β*_3_ × Arm × Period + *b*_0_ + *b*_1_ × Period

where Arm and Period are binary variables for the study arm (full or light intervention) and study period (12-week or 2-year follow-up), respectively; random intercept *b*_0_ and random slope *b*_1_ are included to account for correlation among repeated measures within each individual. The coefficient *β*_1_ indicates the mean outcome difference between the full intervention arm and the light intervention arm at the first 12 weeks; *β*_1_ + *β*_3_ indicates the mean outcome difference between the 2 arms at 2-year follow-up; *β*_2_ indicates the mean outcome change from the first 12-week and 2-year follow-up for the light intervention arm; *β*_2_ + *β*_3_ indicates the mean outcome change between the first 12-week and 2-year follow-up for the full intervention arm; *β*_3_ indicates the difference in mean outcome change from the first 12-week and 2-year follow-up between the full intervention arm and the light intervention arm. The *P* value for testing the significance was calculated based on the estimated coefficient and estimated covariance from the linear mixed effects model.

To compare the trend of adherence and MVPA between the full intervention arm and the light intervention arm, we used the generalized additive mixed effects model (GAMM):

g(y) = *β*_0_ + *β*_1_ × Arm + s(Time) + s(Time) × Arm

where Time is a continuous variable for the study day (day 1, day 2, …); s(Time) is the smooth term for “Time”; and s(Time) × Arm is the interaction term between “Time” and “Arm.” Models with and without prespecified knots were assessed.

We used the minimized generalized cross-validation score for smoothness selection. To select the best fitted model, in terms of the interaction term between time and group and knots specification in the GAMM, we conducted model comparisons using analysis of variance and model’s Akaike information criteria. For the goodness of fit of the chosen models, we examined the model’s deviance and the adjusted R^2^. Graph of the best fit was used to display the trends of adherence and MVPA over the study period.

### Comparison of MVPA Between Preintervention and Postintervention

We also used paired *t* test to compare the MVPA during the preintervention period (measured by ActiGraph) with MVPA during the 12-week intervention, and MVPA during the 2-year follow-up period for both the full intervention arm and the light intervention arm. We also compared the preintervention MVPA between the 2 study groups using the 2-sample independent *t* test (unpaired).

### Ethics Approval

All procedures were approved by the University of California San Diego Human Subjects Protection Program (IRB#140694).

## Results

### Participant Characteristics

Of the 911 women who were screened for eligibility, 97 were eligible and scheduled for a visit, and 87 participants were randomized. Most common reasons for being ineligible were being too active (n=225), unable/unwilling to attend clinic visits (n=106), breast cancer surgery more than 5 years ago (n=81), and medical exclusion (n=36). Of the 87 randomized, 75 agreed to enroll in the 2-year maintenance study: 37/43 in the full intervention arm (86%) and 38/44 in the light intervention arm (86%). The current analyses comprise data from the 75 participants who enrolled in the maintenance study. There were no significant (*P*>.05) differences in demographic or clinical variables between participants who did and did not enroll in the maintenance study.

Participants in the 2-year follow-up study were 75 female breast cancer survivors who were predominantly diagnosed at stage 1. A little more than half had received chemotherapy and at the start of the original trial they were on average 2.6 years from the diagnosis. The average age of participants was 57 years (SD 10.4 years), with the majority being non-Hispanic, White, and having a college education or greater (Table 1). There were no significant (*P*>.05) differences between the 2 arms.

**Table 1:** Baseline characteristics by intervention group.

Characteristics		Full intervention (n=37)	Light intervention (n=38)	All (n=75)
Age (years), mean (SD)		58.2 (11.5)	56.2 (9.1)	57.2 (10.4)
Married status, n (%)		27 (72.9)	27 (71.1)	54 (72.0)
BMI (kg/m^2^), mean (SD)		26.7 (6.4)	27.7 (6.4)	27.2 (6.4)
**Education, n (%)**				
	Some college or less	11 (29.7)	9 (23.7)	20 (26.7)
	College graduate	15 (40.6)	20 (52.6)	35 (46.7)
	Master or higher	11 (29.7)	9 (23.7)	20 (26.7)
**Ethnicity, n (%)**				
	Not Hispanic/Latino	30 (81.1)	33 (86.8)	63 (84.0)
	Hispanic/Latino	7 (18.9)	5 (13.2)	12 (16.0)
**Race, n (%)**				
	White	30 (80.1)	31 (81.6)	61 (81.3)
	Non-White	7 (18.9)	7 (18.4)	14 (18.7)
**Cancer stage, n (%)**				
	Stage 1	22 (59.4)	22 (57.9)	44 (58.7)
	Stage 2	11 (29.7)	13 (34.2)	24 (32.0)
	Stage 3	4 (10.8)	3 (7.9)	7 (9.3)
Received chemotherapy, n (%)		21 (56.7)	20 (52.6)	41 (54.7)
Time since surgery (months), mean (SD)		31.4 (17.0)	30.6 (16.0)	30.9 (16.4)

### Patterns of Adherence to Wearing the Fitbit

**Figure 1:**
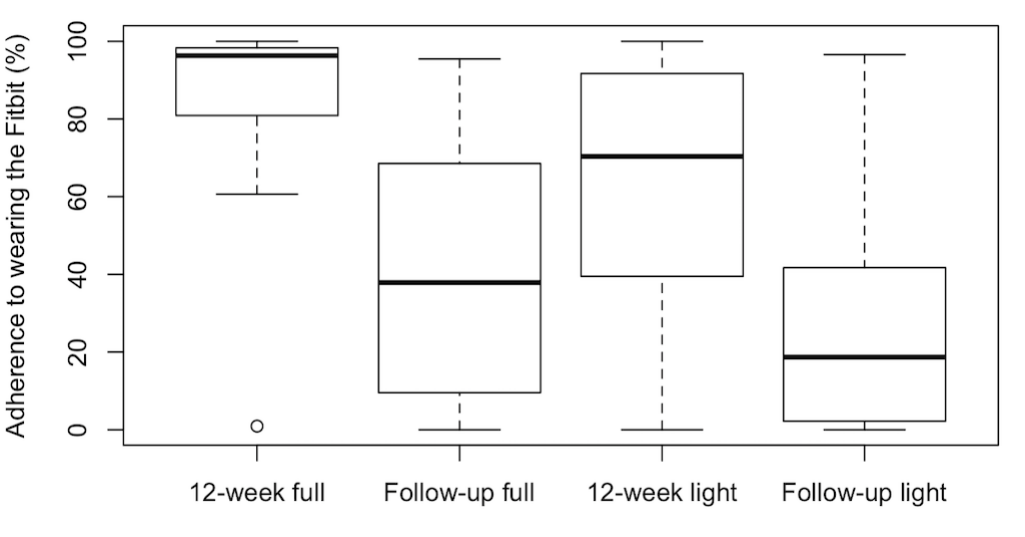
Box-plots of median and interquartile range of adherence to wearing the Fitbit during the 12-week exercise intervention or “light” intervention period for each study group, and during the post-intervention to 2-year follow-up period for each study group.

Average adherence to wearing the Fitbit was significantly higher for the full intervention arm during the 12-week intervention period compared with the light intervention arm during the 12-week intervention period—mean adherence 85% (SD 23%) for the full intervention arm versus 60% (SD 34%) for the light intervention arm (*P*<.001). In addition, average adherence from the postintervention to 2-year follow-up period significantly dropped from the 12-week intervention period for both the full intervention arm (45%, SD 33%; *P*<.001) and the light intervention arm (30%, SD 31%; *P*<.001). However, during the postintervention to 2-year period there were no significant differences in average adherence between the 2 groups (Figure 1)—mean adherence 40% (SD 35%) for the full intervention arm versus 30% (SD 32%) for the light intervention arm (*P*=.71).

**Figure 2:**
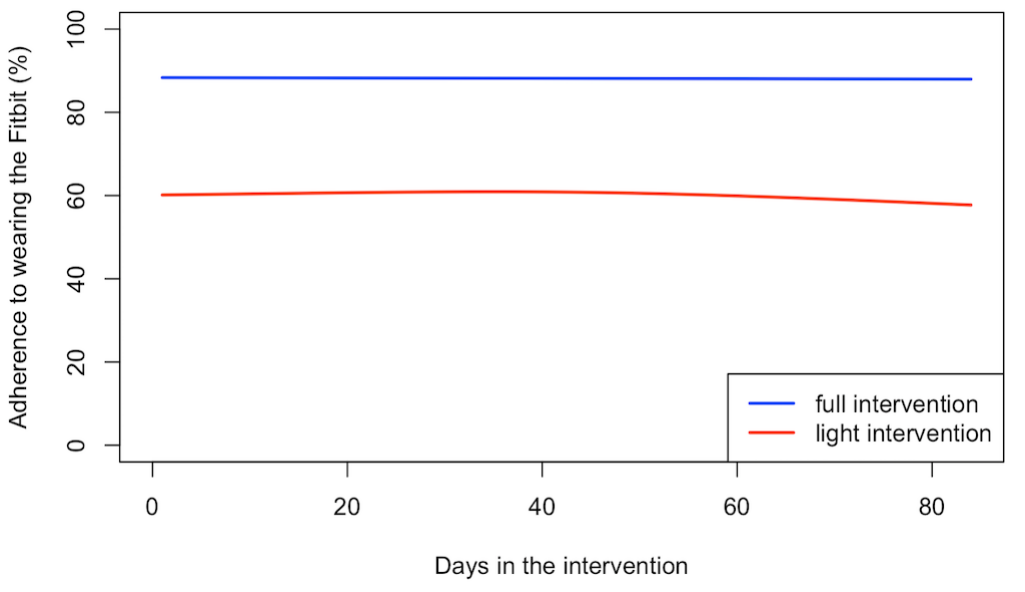
Weekly rolling average adherence to wearing the Fitbit during the 12-week intervention period for the Full Intervention arm and the 12-week “light” intervention period for the Light Intervention arm, by group.

We then compared the temporal patterns of adherence between the 2 groups during the 12-week intervention period and the postintervention to 2-year period using the GAMM. While participants in the full intervention arm had significantly higher (*P*<.001) average adherence during the 12-week interventional period (Figure 2), there was no significant difference in the temporal pattern of adherence across the 12-week period (*P*=.24), with both groups having stable adherence over time.

**Figure 3:**
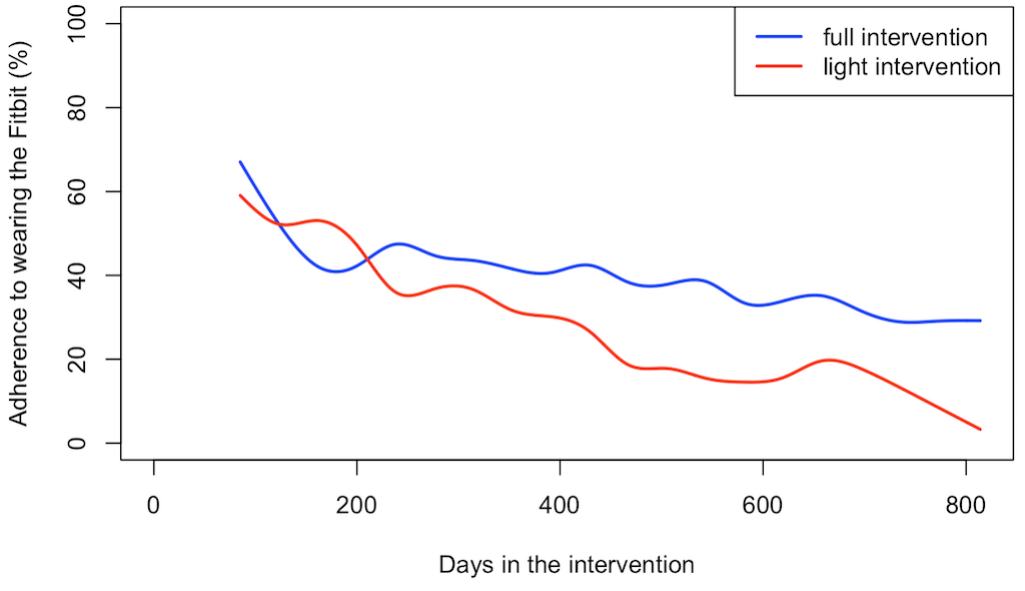
Weekly rolling average adherence to wearing the Fitbit after completion of the 12-week intervention period, by group.

By contrast, in the postintervention to 2-year period (Figure 3), although the average adherence across the entire postintervention period was similar, the daily adherence over time was significantly different between the 2 groups (*P*<.001). While there were steep initial declines in both arms, adherence in the full intervention arm declined more gradually over the remainder of the study period in comparison with the light intervention arm.

A total of 67 participants answered the self-report question of whether or not they were still wearing their Fitbit. Of these, 32 participants reported that they had stopped wearing their Fitbit: 13 (41%) stated that their Fitbit broke, 12 (38%) reported that they lost their Fitbit or their charger, 6 (19%) stated that they were no longer interested in wearing the Fitbit, and 3 (9%) indicated a health issue that stopped them from being active. Several participants replaced lost or broken Fitbits during the follow-up years and then subsequently had a lost or broken Fitbit a second time or lost interest in wearing it.

### Patterns of Fitbit-Measured MVPA

Participants in the full intervention arm significantly increased average minutes of MVPA from preintervention to across the 12-week intervention period (13.95 minutes/week to 27.89 minutes/week; *P*<.001) and participants in the light intervention arm showed a trend for increased average minutes of MVPA from preintervention to across the 12 weeks (14.64 minutes/week to 18.35 minutes/week; *P*=.07; Table 2). Although both arms increased MVPA during the 12-week intervention period, the full intervention arm had significantly higher average minutes of MVPA than the light intervention arm (27.89 minutes/week versus 18.35 minutes/week, respectively; *P*<.001; Table 2). Similar to the adherence results, during the 2-year postintervention period the average MVPA significantly dropped from the 12-week intervention period for both the full intervention arm (21.74 minutes/week at the 2-year follow-up vs 27.89 minutes/week at the 12-week intervention; *P*=.002) and the light intervention arm (15.03 minutes/week at the 2-year follow-up vs 18.35 minutes/week at the 12-week intervention; *P*=.004), but there was no significant difference in average MVPA between the 2 groups (*P*=.33). Although average MVPA decreased during the 2-year follow-up in comparison to preintervention MVPA, there was a trend for greater average MVPA for participants in the full intervention arm (21.74 minutes/week at the 2-year follow-up vs 13.95 minutes/week preintervention; *P*=.08), but no difference from preintervention for the light intervention arm (15.03 minutes/week at the 2-year follow-up vs 14.64 minutes/week preintervention; *P*=.26).

**Table 2:** Minutes per day of moderate-to-vigorous physical activity, by group (N=75).

Physical activity	Full intervention MVPA (minutes/week), mean (SD)	*P* value for comparison of preintervention with postintervention within the full intervention group	Light intervention MVPA (minutes/week), mean (SD)	*P* value for comparison of preintervention with postintervention within the light intervention group	*P* value for comparison between groups
Preintervention (ActiGraph)	13.95 (11.96)	N/A^a^	14.64 (13.46)	N/A	.83
12-week intervention period (Fitbit)	27.89 (16.38)	<.001	18.35 (12.64)	.07	<.001
2-year follow-up (Fitbit)	21.74 (24.65)	.08	15.03 (13.27)	.26	.33

^a^N/A: not applicable.

**Figure 4:**
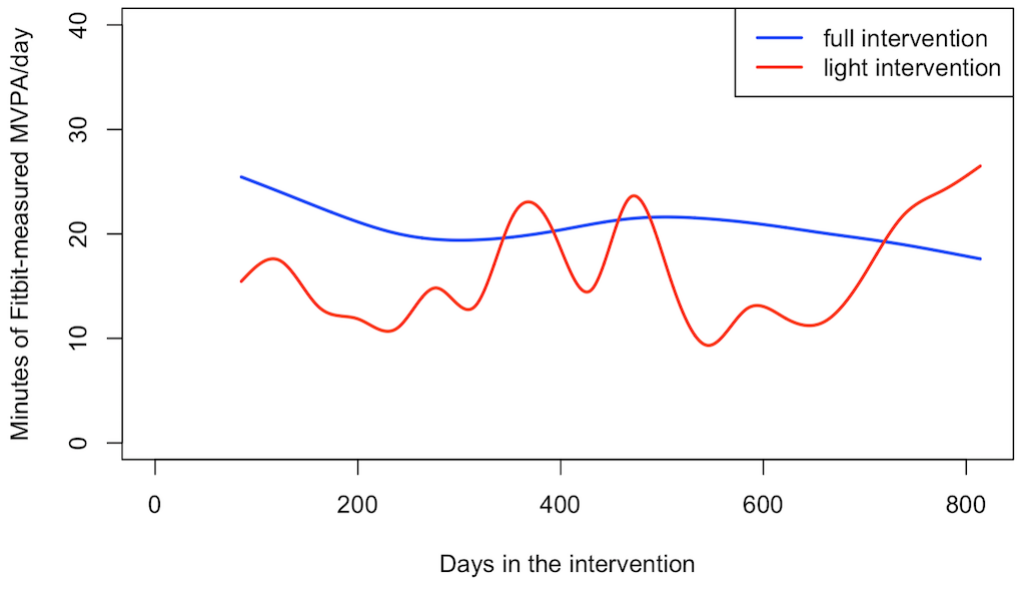
Daily Fitbit measured MVPA during the 12-week intervention period for the Full Intervention arm and the 12-week “light” intervention period for the Light Intervention arm, by group.

We then compared the temporal patterns of activity between the 2 groups during the 12-week intervention period and from the postintervention to 2-year period (Figure 4). Similar to the adherence results, while the full intervention arm had significantly higher (*P*=.002) daily MVPA than the light intervention arm during the intervention, there was no difference in the temporal pattern of daily MVPA across the 12-week period (*P*=.99), with both groups having relatively stable daily MVPA.

**Figure 5:**
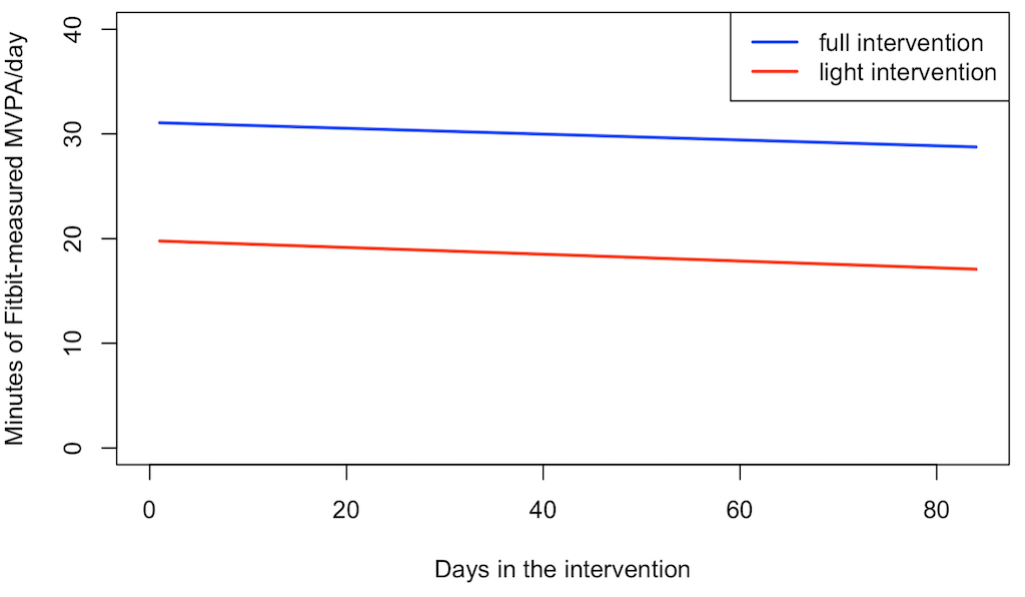
Daily Fitbit measured MVPA after completion of the 12-week intervention period, by group.

While the average MVPA for the entire postintervention period was similar between groups, the daily MVPA over time was significantly different between the 2 groups (Figure 5; *P*<.001). Among participants who continued to adhere to wearing the Fitbit, the full intervention arm had a relatively stable trend with a gradual decline in daily MVPA, while the light intervention arm had an irregular temporal pattern with fluctuations in MVPA over time. Of note, with the relatively low level of adherence that continued to decline over the follow-up period, the curvature trend of the daily MVPA in the light intervention arm was measured in a small number of individuals.

## Discussion

### Principal Findings

This study examined patterns of wearing an activity tracker and engaging in MVPA during and after completion of a 12-week randomized trial of a full exercise intervention in comparison with a light intervention, through 2 years of follow-up. Our study yielded several key findings. First, the exercise intervention, which entailed more comprehensive feedback and external accountability, resulted in greater adherence to wearing the Fitbit and minutes of MVPA than the light intervention. Second, both full and light interventional groups had significant reductions in adherence and physical activity during the long-term follow-up. Finally, while both groups had similar average adherence and MVPA during the postintervention to 2-year period, the full intervention group had a more stable temporal pattern of adherence and daily MVPA during this time than the light intervention group, in addition to a trend toward maintaining some gains in MVPA over preintervention levels. These results provide new insight into wearable technology and activity patterns during and after completion of an exercise intervention and suggest the potential importance of sustained self-monitoring and feedback interventions to maintain increased activity levels over time.

We found that the full exercise intervention resulted in greater daily MVPA in comparison with the light intervention during the 12-week interventional period. The primary added feature of the full exercise intervention was external accountability, where participants were aware that their activity would be checked by their health coach, discussed with them at planned phone calls, and would receive additional contacts in between calls based on their Fitbit data. Our results suggest that this accountability led to greater adherence to wearing the Fitbit and MVPA during the intervention period. Wearing the Fitbit did not decrease over time during the 12-week intervention period for either study arm. This may have been due to the external cues and reminders to wear and sync their Fitbit that participants received from the emails that came 2-3 times a week during that period, but stopped at 12 weeks, or may have been due to the novelty of using a Fitbit. However, wearing the Fitbit decreased after the intervention period with the most commonly reported reasons for discontinued use of the Fitbit were that it broke or was lost. Now that most Fitbits are wrist-worn, it may help to decrease loss of devices, but devices breaking is likely to be a continued issue that impacts continued wearing of devices. With the well-established benefits of self-monitoring for behavior change, identifying ways to increase long-term engagement with activity trackers is needed.

We also found that both the full and the light intervention group increased minutes of MVPA from the preintervention to the intervention period and maintained it during the 12-week intervention period. The initial increase in MVPA at the start rather than gradually increasing overtime may have been due to the intervention’s goal-setting approach that utilized motivational interviewing, where participants were allowed to set any starting goal that they chose. With more studies having day-level physical activity data, future studies could examine different methods of setting goals and different patterns of exercise to see if they relate to long-term maintenance of activity. This study adds to the literature by demonstrating the importance of additional intervention components, particularly increasing external accountability, when using activity trackers to promote exercise, and the challenges with lost and broken trackers.

Our study is one of the first to use wearable activity trackers to assess long-term maintenance of behavior after completion of a short-term intervention in breast cancer survivors. Although some prior studies have assessed long-term physical activity, they have either examined sustained long-term physical activity interventions or relied upon self-reported MVPA [9]. In our study, there were significant declines in wearing the Fitbit and activity levels after the end of the intervention. This is consistent with previous studies in cancer survivors that have generally found that activity levels reduce from the end of the interventional period [9]. Our results suggest that simply allowing participants to keep a wearable tracker is not sufficient to maintain activity levels in the long term. As the novelty of having the tracker wanes, additional measures, such as continued external accountability or coaching, may be beneficial.

Although there were significant declines in activity levels during the postintervention follow-up for both groups, it is interesting that the temporal patterns of both adherence and physical activity were more stable in those who received the full exercise intervention. Comparison of these results with previous trials is difficult as this study took advantage of having minute-level physical activity data for months, rather than having brief snapshots of MVPA from 7 days of accelerometer wear or self-report. By examining patterns of activity over time we see that even a short-term intensive exercise intervention may result in some lasting change in behavior patterns beyond the intervention period. Together, the results suggest that an intensive short-term physical activity intervention, coupled with a continued long-term maintenance intervention, may be necessary to sustain higher activity levels in the long term. Further study is needed to develop the optimal short- and long-term strategies to enhance activity tracker use to achieve sustained physical activity.

### Limitations

This study provides unique insight into long-term activity levels after completion of an exercise intervention, but there are several limitations that should be noted. The sample size limited our ability to detect potentially smaller differences between groups, including average adherence and MVPA in the postintervention period. In addition, the progressive decline in adherence to wearing the Fitbit in long-term follow-up meant that there was a large amount of missing MVPA data. Without other measures of MVPA we are unable to know how much activity individuals were engaging in after their Fitbit broke, was lost, or if they were no longer interested in wearing, and our results are limited to those who continued to wear their Fitbit. In addition, the predominantly well-educated, White non-Hispanic sample may limit external generalizability. The sample also had a majority of early stage breast cancer survivors and thus may not generalize to women with more advanced breast cancer. Although the initial trial was randomized, there is the potential for selection bias among those participants who decided to continue in the long-term study. Finally, knowledge of participation in the study may have conferred some effect of external accountability among participants that would not be present outside of the research setting.

### Conclusions

This study examined patterns of wearable technology use and activity levels among breast cancer survivors during and after completion of a physical activity intervention. We found higher activity levels among participants receiving an intervention with greater engagement and accountability, but that activity levels reduced in follow-up after completion of the intervention. These results provide important insights regarding behavior during and after a physical activity intervention, and may help inform the design of future interventions to more effectively promote, both short- and long-term, sustained increases in physical activity.

## Abbreviations

GAMM: generalized additive mixed effects model
MET: metabolic equivalents
MVPA: moderate-to-vigorous physical activity
